# Comparison of IVA and GIG-ICA in Brain Functional Network Estimation Using fMRI Data

**DOI:** 10.3389/fnins.2017.00267

**Published:** 2017-05-19

**Authors:** Yuhui Du, Dongdong Lin, Qingbao Yu, Jing Sui, Jiayu Chen, Srinivas Rachakonda, Tulay Adali, Vince D. Calhoun

**Affiliations:** ^1^The Mind Research NetworkAlbuquerque, NM, USA; ^2^School of Computer and Information Technology, Shanxi UniversityTaiyuan, China; ^3^Brainnetome Center and National Laboratory of Pattern Recognition, Institute of Automation, Chinese Academy of SciencesBeijing, China; ^4^Department of Computer Science and Electrical Engineering, University of Maryland Baltimore CountyBaltimore, MD, USA; ^5^Department of Electrical and Computer Engineering, University of New MexicoAlbuquerque, NM, USA

**Keywords:** functional magnetic resonance imaging (fMRI), brain functional networks, independent component analysis (ICA), group information guided ICA (GIG-ICA), independent vector analysis (IVA)

## Abstract

Spatial group independent component analysis (GICA) methods decompose multiple-subject functional magnetic resonance imaging (fMRI) data into a linear mixture of spatially independent components (ICs), some of which are subsequently characterized as brain functional networks. Group information guided independent component analysis (GIG-ICA) as a variant of GICA has been proposed to improve the accuracy of the subject-specific ICs estimation by optimizing their independence. Independent vector analysis (IVA) is another method which optimizes the independence among each subject's components and the dependence among corresponding components of different subjects. Both methods are promising in neuroimaging study and showed a better performance than the traditional GICA. However, the difference between IVA and GIG-ICA has not been well studied. A detailed comparison between them is demanded to provide guidance for functional network analyses. In this work, we employed multiple simulations to evaluate the performances of the two approaches in estimating subject-specific components and time courses under conditions of different data quality and quantity, varied number of sources generated and inaccurate number of components used in computation, as well as the presence of spatially subject-unique sources. We also compared the two methods using healthy subjects' test-retest resting-state fMRI data in terms of spatial functional networks and functional network connectivity (FNC). Results from simulations support that GIG-ICA showed better recovery accuracy of both components and time courses than IVA for those subject-common sources, and IVA outperformed GIG-ICA in component and time course estimation for the subject-unique sources. Results from real fMRI data suggest that GIG-ICA resulted in more reliable spatial functional networks and yielded higher and more robust modularity property of FNC, compared to IVA. Taken together, GIG-ICA is appropriate for estimating networks which are consistent across subjects, while IVA is able to estimate networks with great inter-subject variability or subject-unique property.

## Introduction

There is a rapidly increasing interest in using functional magnetic resonance imaging (fMRI) data to characterize brain functional networks. Independent component analysis (ICA), a data-driven method, has been widely used to analyze fMRI data without requiring the definition of brain regions (or nodes). Spatial ICA (sICA) (McKeown et al., 1998), a popular method for analyzing fMRI data, decomposes fMRI data into a linear mixture of spatially independent components (ICs) some of which are subsequently identified as brain functional networks. Despite success of ICA in fMRI data analyses, ICA faces some challenges. The order of resulting ICs from individual-subject ICA is arbitrary, increasing the difficult to establish correspondence among ICs estimated from different subjects. Another issue is that the estimated ICs include not only meaningful functional networks, but also various artifact-related ICs resulting from imaging and non-neural physiological activity. In addition, the number of sources is unknown, so the number of ICs always needs to be estimated (Li et al., 2007); however the numbers estimated using different criteria are varied (Zuo et al., 2010). These shortcomings of ICA bring difficulties to multiple-subject fMRI data analyses, especially when shared networks across subjects are expected for subsequent group analyses. To address the problems, group independent component analysis (GICA) and independent vector analysis (IVA) have been proposed.

Several GICA frameworks have been proposed for fMRI data analyses, including using the spatial concatenation (Svensén et al., 2002), temporal concatenation (Calhoun et al., 2001, 2009; Beckmann et al., 2009) and tensor organization (Beckmann and Smith, 2005) strategies. Relative to ICA on each individual-subject's data, one advantage of GICA is that it can build direct correspondence of ICs across subjects. Among the GICA approaches, the temporal concatenation based method is most widely used. This approach first estimates the group-level ICs by performing ICA on the time points-concatenated fMRI data of all subjects, and then back-reconstructs the subject-specific ICs mainly using principal component analysis (PCA) based (Calhoun et al., 2001; Erhardt et al., 2011) or regression based (Beckmann et al., 2009; Erhardt et al., 2011) algorithms. More recently, in order to improve the accuracy of the subject-specific ICs estimation, group information guided independent component analysis (GIG-ICA) (Du and Fan, 2013; Du et al., 2015c, 2016a) as a variant of GICA has been proposed. GIG-ICA estimates the subject-specific ICs under the guidance of the group-level ICs by using a multi-objective function optimization framework, which simultaneously optimizes the independence among multiple ICs of each subject and the correspondence between each group-level IC and the associated subject-specific IC. The optimization of independence of multiple components for each subject's data benefits yielding accurate subject-specific functional networks. The optimization of correspondences between one group-level IC and the associated subject-specific ICs guarantees that the obtained individual networks have the same physiological meanings and then are comparable across subjects. Therefore, compared to the traditional PCA-based and regression-based back-reconstruction techniques that ignore independence of individual ICs to some extent, GIG-ICA can yield more accurate individual networks and the associated time courses (Du and Fan, 2013; Du et al., 2016a) while still preserving correspondence and comparability of shared networks across different subjects. Notably, GIG-ICA can estimate individual networks for new data by utilizing *a prior* spatial network maps as guidance. Our previous work (Du et al., 2015c) has shown the promise of GIG-ICA to estimate spatial functional networks from fMRI data. By applying GIG-ICA to resting-state fMRI data (Du et al., 2014, 2015c), we found potential biomarkers in several functional networks for distinguishing schizophrenia, bipolar disorder and schizoaffective disorder. In addition, GIG-ICA (Du et al., 2015b, 2017) has the ability to extract functional connectivity states from time-varying functional connectivity (Calhoun et al., 2014; Du et al., 2015a, 2016b). Our work (Du et al., 2015b, 2017) revealed interesting biomarkers of schizophrenia, bipolar disorder and schizoaffective disorder in multiple connectivity states. In this paper, we only focus on the application of GIG-ICA in estimating functional networks from fMRI data.

Independent vector analysis (IVA), an alternative method to achieve an independent decomposition (Adali et al., 2014), has been applied to analyzing fMRI data of schizophrenia patients (Gopal et al., 2016) as well as patients with stroke (Laney et al., 2015a,b). The approach models both the independence of individual components and the dependence of similar components across subjects. Several advancements of IVA have been made for achieving reliable source separation for linearly dependent Gaussian and non-Gaussian sources (Anderson et al., 2010, 2014; Dea et al., 2011; Li et al., 2011; Adali et al., 2014; Boukouvalas et al., 2015). Among those, IVA-GL (IVA with multivariate Gaussian source component vectors plus IVA with Laplace source component vectors), which is a combination of two IVA algorithms, IVA with multivariate Gaussian component vectors (IVA-G) (Anderson et al., 2012) and IVA with multivariate Laplace component vectors (IVA-L) (Lee et al., 2008), provides an attractive tradeoff in terms of complexity and performance and has been the algorithm used in previous applications of IVA to fMRI data (Laney et al., 2015a,b; Gopal et al., 2016). Previous studies (Dea et al., 2011; Ma et al., 2013; Michael et al., 2014; Laney et al., 2015a,b) compared IVA and traditional GICA under different levels of subject variability and parameters, and showed outperformance of IVA over GICA in terms of capturing subject-specific variability.

Both IVA and GIG-ICA are able to optimize the independence among intra-subject components and dependence among inter-subject components, and showed advantages over traditional GICA in several comparison studies (Dea et al., 2011; Du and Fan, 2013; Ma et al., 2013; Michael et al., 2014; Du et al., 2016a). However, a full comparison between IVA and GIG-ICA has not been well studied, especially in neuroimaging application. In this paper, we compare their performance using both simulations and real fMRI data. We evaluate the two methods with respect to the estimation accuracy of components and time courses by using simulated data with different quality and quantity, data with varied number of sources generated, inaccurate number of components for computation, as well as data with subject-unique sources. In addition, test-retest resting-state fMRI datasets are also utilized to compare the two methods in terms of estimated functional networks and interaction among networks. We assess if IVA and GIG-ICA can yield reliable network maps and functional network connectivity (FNC) using the test-retest data. With these detailed comparisons, we expect to gain more knowledge of both methods in analzying fMRI data under different scenarios and thus provide guidance for researchers in the field.

## Materials and methods

### IVA and GIG-ICA

As for IVA, IVA-GL algorithm was adopted to estimate components for comparisons in this work. It can be accessed in Group ICA for fMRI toolbox (GIFT) (http://mialab.mrn.org/software/gift/index.html). There are mainly two steps: (1) performing subject-level PCA on each subject's data; (2) applying IVA-GL to estimate the subject-specific components and time courses (TCs). The estimated components are then Z-scored. A free parameter is the number of components used for the subject-level PCAs, denoted as *I*1.

GIG-ICA (Du and Fan, 2013; Du et al., 2016a), also included in GIFT, involves the following steps: (1) performing subject-level PCA reduction on each subject's data and group-level PCA on the temporal concatenation of subject-level PCAs reduced data; (2) applying group-level ICA to the reduced data using Infomax algorithm (Bell and Sejnowski, 1995); (3) identifying and removing artifact-related group-level ICs; (4) computing each subject-specific IC via a multi-objective function optimization based on the individual-subject data and each remaining non-artifact group-level IC (Du and Fan, 2013) using a deflation manner; and finally (5) estimating the subject-specific TCs. In step (4), GIG-ICA simultaneously optimizes the independence of each subject-specific IC, measured by negentropy, as well as the correspondence between each subject-specific IC and each group-level IC, measured by their correlation (Du and Fan, 2013), automatically resulting in Z-scored subject-specific ICs. Relevant parameters include the number of principal components (PCs) used for the subject-level PCAs, denoted as *G*1, and the number of PCs/ICs used for the group-level PCA/ICA, denoted as *G*2.

It is worth noting that in order to decrease computation load, GIG-ICA can remove artifact-related group-level ICs before estimating individual components (Du et al., 2016a), only yielding subject-specific meaningful networks. However, IVA has to compute all components and remove artifact-related components in a subsequent postprocessing stage. To facilitate comparison between GIG-ICA and IVA, we computed all components in GIG-ICA without performing artifact removal after the group-level ICA step. In experiments using real fMRI data, we matched components between the two methods after obtaining the individual results and then removed the corresponding artifact-related components for both methods. For comparison, we also set *G*1 = *G*2 = *I*1, resulting in equivalent numbers of components for the two methods. In this work, Infomax algorithm employed in the first step (i.e., the group-level ICA) of GIG-ICA and IVA-GL algorithm are comparable, since both methods use fixed nonlinearity matched to super-Gaussian sources.

### Experiments using simulations

Due to that there is no ground truth in practical applications, simulation-based tests are necessary for evaluating different methods. In order to comprehensively compare IVA and GIG-ICA, we performed several experiments to assess the accuracy of the estimated individual components and TCs under different conditions, including various data quality and quantity (Experiment 1), varied number of sources and inaccurate number of components for computation (Experiment 2), and spatially subject-unique sources (Experiment 3). In each experiment, we simulated fMRI-like data of multiple subjects using the SimTB toolbox (Allen et al., 2012; Erhardt et al., 2012). The number of subjects *M* was simulated to be 10. For each subject, *C* source images (148 × 148 pixels) and their corresponding TCs (150 or less time points in length) were simulated and then used to generate data by a linear mixture model. In our experiments, we set *C* to be 7 or 8. Rician noise was then added to data with a specified contrast-to-noise ratio (CNR). Repetition time (TR) was 2 s/sample. Among *C* sources, some sources were similar across all subjects with slight variance (i.e., subject-common), while the other sources were unique and only present on specific subject (i.e., subject-unique). These subject-unique sources were generated to simulate significant source variability across subjects. The parameters of our experiments are summarized in Table 1.

**Table 1:** **Parameters of simulations and methods in the following simulation-based experiments**.

	**Experiment 1**		**Experiment 2**		**Experiment 3**
	**Different data quality**	**Different data quantity**	**Varied number of sources generated**	**Inaccurate number of components used in computation**	**Spatially subject-unique source**
Number of subjects	10	10	10	10	10
Number of sources in each subject's data	8	8	8 (*i* = 1, ⋯ , 5), 7 (*i* = 6, ⋯ , 10), *i* denotes the subject index	8	8
CNR in each subject's data	0.5–2 with the step of 0.1	2	2	2	2
Number of time points in each subject's data	150	40–120 with the step of 20	150	150	150
Source type	similar across subjects	similar across subjects	similar across subjects	similar across subjects	one subject-unique source
Number of components used in computation	8	8	7 and 8	6, 8, and 10	8

#### Experiment 1: comparing IVA and GIG-ICA using data with different quality and quantity

**Figure 1:**
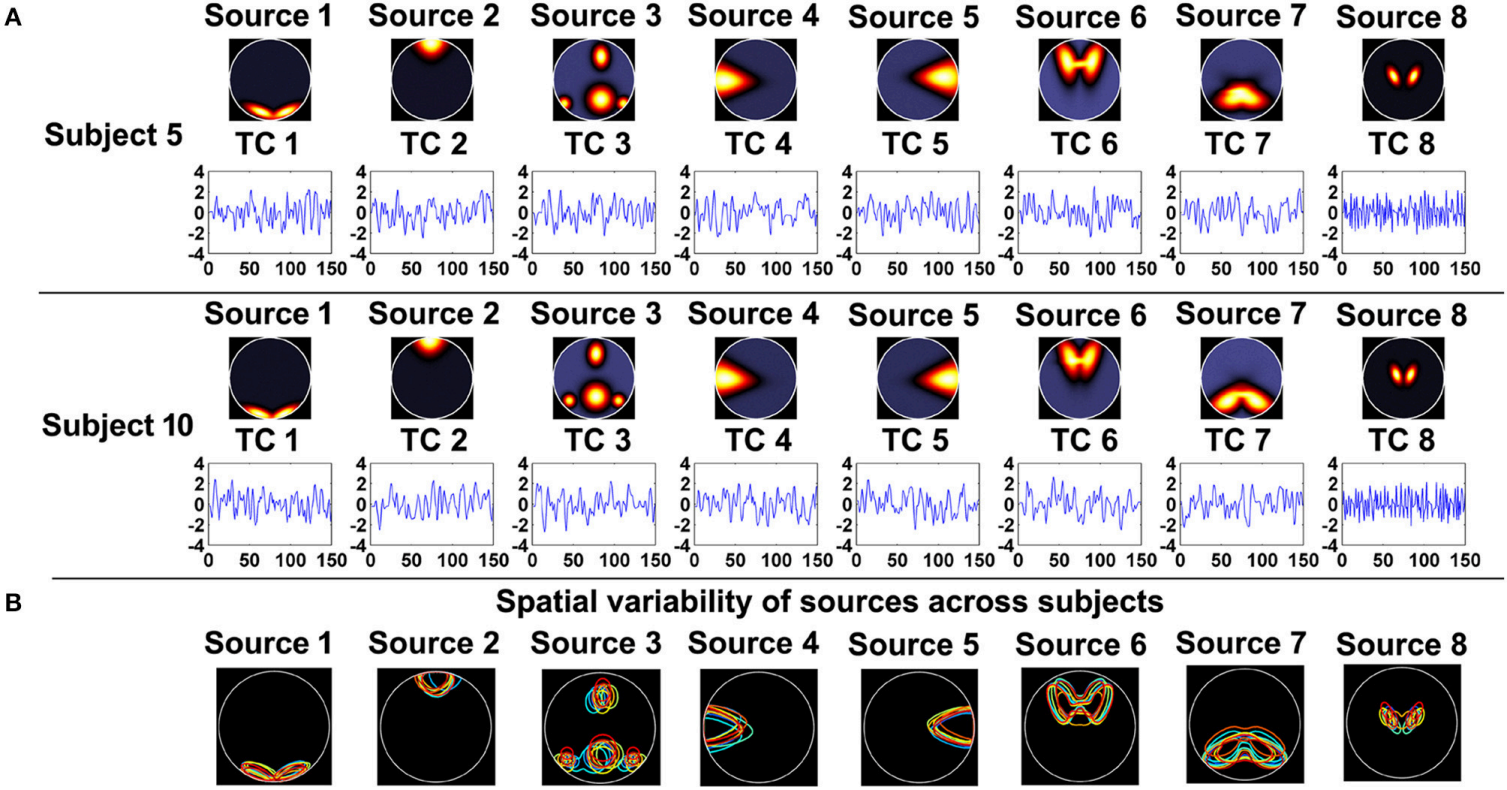
**(A)** The simulated sources and their associated time courses (TCs) of two subjects in Experiment 1. **(B)** The spatial variability of sources across subjects. Each color denotes the source contours of a different subject.

As shown in Figure 1A, 8 sources and their associated TCs were simulated for each subject. Similar to previous work, each of the 8 sources was generated from a common map with added spatial variability across subjects by random translations [mean = 0 pixel; standard deviation (SD) = 5 pixels], rotations (mean of 0 degree; *SD* = 3°), and spatial extents (i.e., spreads) of the common spatial map (mean = 3 magnification; *SD* = 0.03 magnification). Thereby, sources were spatially consistent across subjects but showing moderate subject-specific variability, as shown in Figure 1B. Additionally, temporal variation was applied in simulation of TCs. In order to evaluate the effect of data quality, for each subject, we simulated 16 datasets with different CNRs (ranging from 0.5 to 2 with the step of 0.1) and a fixed number of time points (i.e., 150). Subsequently, regarding each specific CNR (e.g., the CNR = 1), we performed IVA and GIG-ICA on the associated data of all subjects, respectively. To investigate the effect of data quantity, for each subject, we simulated 5 datasets by varying the time points from 40 to 120 in steps of 20 while the CNR was fixed at 2. Afterwards, in terms of each given number (e.g., the number of time points = 100), each method (IVA or GIG-ICA) was applied to the relevant data of all subjects. For these experiments, we set the number of components (i.e., *G*1, *G*2, *I*1) used in the analyses to be the same as the number of true sources (i.e., 8).

#### Experiment 2: comparing IVA and GIG-ICA under conditions of varied number of sources and inaccurate number of components

In this section, we first assessed the performance of the two methods using data with varied number of sources across different subjects (see Table 1 for the detailed parameters). Five subjects' datasets were simulated with 8 sources, while the other five subjects' datasets were simulated with 7 sources. Among the sources, each of 7 sources had a similar spatial pattern across all subjects with slight inter-subject variability, while the other source was only present in five subjects with small spatial variation. Considering the difference in the simulated number of sources across different subjects, we performed two comparisons by setting the same number of components in IVA and GIG-ICA to 7 and 8 separately.

It is known that prior to ICA, the number of components is a free parameter, typically either selected by the user or estimated by some information-based criteria (Li et al., 2007; Fu et al., 2014). This parameter may influence the source decomposition since the measure of total component independence may change and thus converge to different solution. In order to evaluate the effect of an inaccurate component number in both methods, based on the data generated with 8 sources (i.e., the data from Experiment 1 with the CNR = 2) we examined each method by setting the number of estimated components to 6, 8, and 10, respectively.

#### Experiment 3: comparing IVA and GIG-ICA using data with spatially subject-unique sources

**Figure 2:**
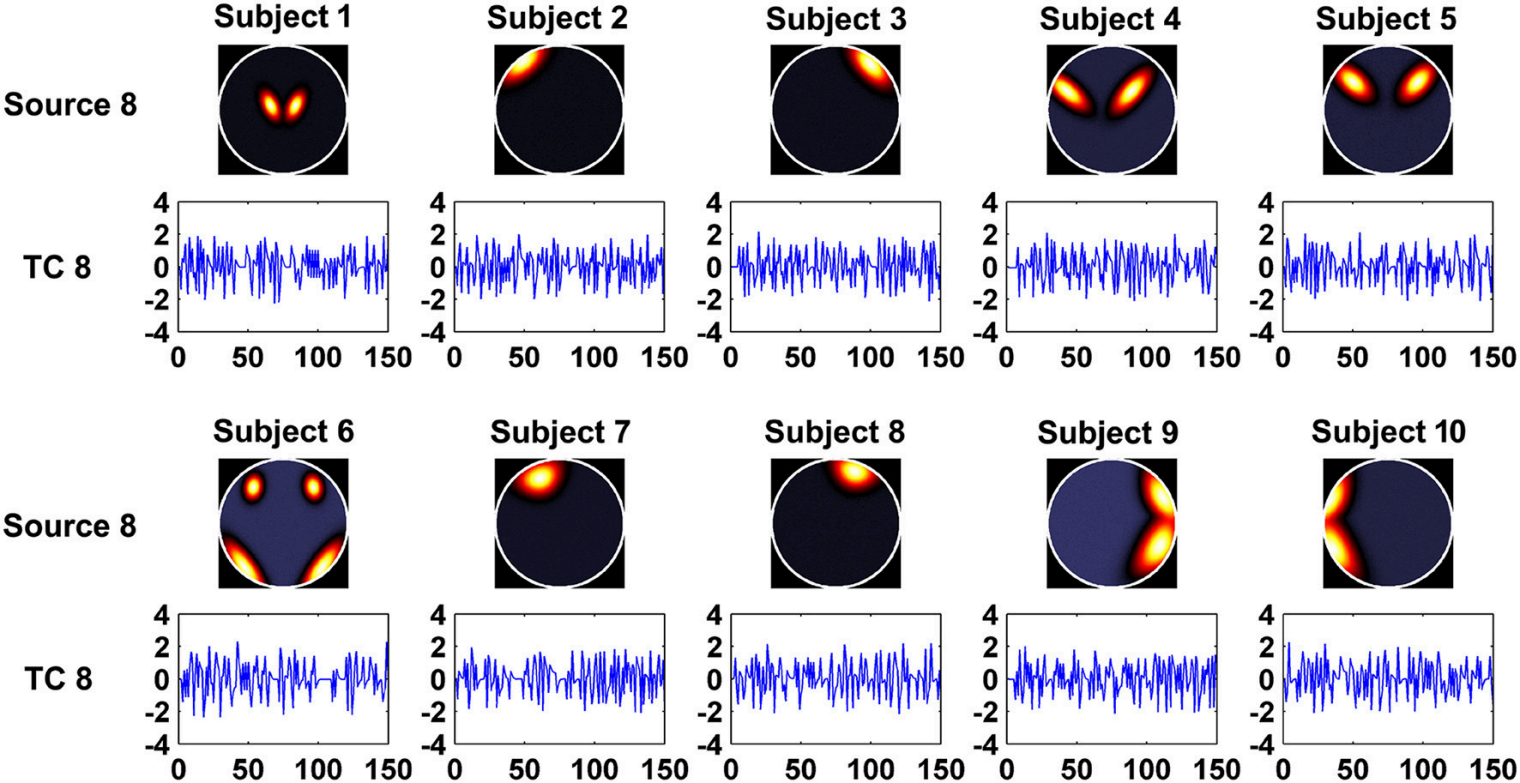
**The simulated subject-unique sources (the 8th sources) and related TCs of all subjects in Experiment 3**.

In this experiment, we aimed to evaluate the ability of the two methods in recovering both subject-common and subject-unique sources. Each subject's data was simulated by using 7 sources each of which was similar across subjects and one additional source unique for each individual subject. The 7 sources had similar patterns with the first 7 sources generated in Experiment 1. Figure 2 shows the simulated subject-unique sources (i.e., the 8th sources) and related TCs of all subjects. The number of components used in computation was specified as the real number of sources (i.e., 8).

#### Evaluation metrics in simulation-based experiments

To compare the performance of IVA and GIG-ICA on simulations, we evaluated accuracy of the estimated subject-specific components and TCs using correlation between estimation and ground truth, consistent to many prior studies (Schmithorst and Holland, 2004; Allen et al., 2012; Du and Fan, 2013; Michael et al., 2014; Du et al., 2016a). We firstly matched the estimated subject-specific components with the simulated subject-specific ground-truth (GT) sources as follows. Regarding each source in Experiment 1 and 2, the corresponding GT sources of all subjects were averaged, and then the mean GT sources were used as source templates. Next, for GIG-ICA method, we matched the group-level ICs with the source templates using a greedy rule (see the Supplementary Material for details). Similarly, for IVA method, we averaged the corresponding components from all subjects to represent the group-level components, which were then matched with the sources templates. For each method, based on the matched results between the group-level components and the source templates, the estimated subject-specific components/TCs were then accordingly matched to the subject-specific GT sources/TCs. For Experiment 3 which involved a subject-unique source in data, we first averaged each of 7 subject-common GT sources across subjects to get its source template, and then matched the 8 group-level component maps obtained from each method with the 7 source templates, consequently constructing correspondence between 7 components and 7 GT sources for those subject-common sources of each subject. Thus, one additional subject-unique component can be matched to the subject-unique GT source for each subject. After matching, we computed the absolute value of Pearson correlation coefficient between each estimated component/TC and its matched GT source/TC to measure the component/TC accuracy. In Experiment 1 and 2, we further calculated the mean of all components/TCs accuracy measures of each subject to reflect its overall component/TC accuracy. In Experiments 1 and 2, for each setting, a two-tailed paired *t*-test was performed to compare the overall component (or TC) accuracy metrics of all subjects from IVA with that from GIG-ICA. In Experiment 3, for each component, we compared the spatial (or temporal) accuracy of all subjects between IVA and GIG-ICA using one two-tailed paired *t*-test. The results were corrected using *p* < 0.05 with Bonferroni correction.

### Experiments using test-retest resting-state fMRI data

Seventy five resting-state fMRI datasets (Zuo et al., 2010) comprising 25 healthy participants with three scans were adopted in the experiment. Each dataset consisted of 197 contiguous EPI functional volumes (*TR* = 2,000 ms; *TE* = 25 ms; flip angle = 90°, 39 slices, matrix = 64 × 64; FOV = 192 mm; acquisition voxel size = 3 × 3 × 3 mm). The first scan (scan 1) is in a scan session. Five to Sixteen months (mean 11 ± 4) after scan 1, scan 2 and scan 3 were conducted with short interval (about 45 min). The fMRI images were preprocessed using SPM8 (http://www.fil.ion.ucl.ac.uk/spm). The first 10 images were discarded, and the remaining 187 images were slice-time corrected and realigned to the first volume for head-motion correction. Subsequently, the images were spatially normalized to the Montreal Neurological Institute (MNI) EPI template and spatially smoothed with a 6 mm FWHM Gaussian kernel.

IVA and GIG-ICA were applied to all 75 preprocessed datasets, respectively, to estimate brain functional networks and their associated TCs of each dataset. For a comprehensive evaluation of these two methods, we used both low and high numbers of components for analyses. When a low number is used, it makes more sense to think of each meaningful component itself as a brain functional network. Many studies (Meda et al., 2014; Du et al., 2015c) have conducted analyses on spatial maps of networks revealed by ICA with low model order, aiming to explore disease biomarkers. In contrast, if a high number is used, the meaningful networks were then usually used as nodes for computing consequent FNC (Allen et al., 2011). Each FNC matrix, which is computed based on the individual-subject's TCs of networks, reflects interaction among different networks. To be consistent with previous studies (Allen et al., 2011; Du and Fan, 2013; Du et al., 2015c, 2016a), we specified 20, 25, and 30 as low model order settings, and 75 and 100 as high model order settings. For simplification, we assessed the results from both low and high model orders using the same manner by considering properties of both networks' spatial maps and interaction among networks (i.e., FNC). Regarding results from each model order setting, we first matched the obtained components from the two methods based on their group-level component maps using a greedy rule (see the Supplementary Materials). Then, based on the matched components with high similarity (correlation > 0.5) between the two methods, we only selected the meaningful networks by manually inspecting spatial and temporal information of the matched components (Allen et al., 2014; Du et al., 2016a) for further investigation. Next, the following evaluations in terms of network maps and FNC were performed on the selected networks for each method. Finally, the performances of the two methods under different model orders were compared.

For each selected network, we evaluated its reliability based on the estimated individual networks from 75 datasets as follows, which is consistent to previous studies (Zuo et al., 2010; Du et al., 2016a). First, voxel-wise right-tailed one-sample *t*-tests (*p* < 0.01 with false discovery rate (FDR) correction) were performed on the corresponding networks of all 75 datasets. Next, since the data from scan 2 and scan 3 were collected with short intervals, voxel-wise intra class coefficients (ICCs) (Zuo et al., 2010) between the corresponding 25 networks from scan 2 and the corresponding 25 networks from scan 3 were calculated to assess the short-term reliability of the network, resulting one 3D ICC map reflecting short-term reliability of the network. In our work, ICC of each voxel was computed using a model (Zuo et al., 2010; Guo et al., 2012) based on one-way analysis of variance (ANOVA), due to that those subjects were scanned using the same scanner. The used equation was: $ICC=\frac{\sigma_{p}^{2}}{\sigma_{p}^{2}+\sigma_{\text{e}}^{2}}$, where $\sigma_{p}^{2}$ denotes the variance of inter-subject effect and $\sigma_{\text{e}}^{2}$ denotes the variance of measurement error. As mentioned above, the data of scan 2 and scan 3 were collected after several months of scan 1. So, we computed ICCs between the corresponding 25 networks from scan 1 and the averaged 25 networks from scan 2 and scan 3 to assess the long-term reliability of the network, resulting in one 3D ICC map reflecting long-term reliability. Based on each ICC map reflecting short-term or long-term reliability of the network, the ICC values were then averaged across voxels within a specific mask which included statistically significant voxels for both methods based on the one-sample *t*-tests results after FDR correction, to summarize the short-term or long-term reliability of the network.

To investigate network interaction, we calculated FNC for each of the 75 datasets, and then evaluated graph-theory based measures using the brain connectivity toolbox (https://sites.google.com/site/bctnet/) as well as reliability in both connectivity and modularity. First, for each dataset, we obtained one FNC matrix by computing Pearson correlation coefficients between the associated TCs of any paired networks. Next, we averaged the FNC matrix across all 75 datasets. Based on the mean FNC matrix, we detected its modules (i.e., network communities) using the most applied eigenvector-based method (Newman M. E., 2006; Newman M. E. J., 2006), where the modularity *Q*-value reflects the accuracy or quality of a community structure. Greater *Q*-value represents stronger modular structure. Subsequently, modularity analysis was also performed on each individual FNC matrix, resulting in a module segmentation and related *Q*-value for each dataset. Since different datasets may have greatly varied modular brain networks, we measured modularity similarity between any pair of datasets using the adjusted mutual information (AMI), consistent to a recent study (Liao et al., 2017). The mean of AMI values computed between datasets in scan 2 and datasets in scan 3 was used to measure the short-term modularity reliability. The mean of AMI values obtained between datasets in scan 1 and datasets in scan 2 or 3 was used to reflect the long-term modularity reliability. Additionally, each connectivity's short-term and long-term reliability in FNC was examined using ICC. Specifically, for each connectivity (i.e., one element in FNC matrix), ICC between the corresponding 25 connectivity strengths from scan 2 and the corresponding 25 connectivity strengths from scan 3 was calculated to assess the short-term reliability of the connectivity; ICC between the corresponding 25 connectivity strengths from scan 1 and the corresponding 25 connectivity strengths averaged between scan 2 and 3 was calculated to assess the long-term reliability of the connectivity. Finally, we calculated the averaged node strength, clustering coefficient, global efficiency, and local efficiency (Rubinov and Sporns, 2010) based on each individual FNC matrix, the elements of which were first changed to their absolute values and thresholded to preserve half elements with higher values (sparsity = 0.5) (Du et al., 2016b).

## Results

### Results from simulation-based experiments

#### Component and time course accuracy estimated from IVA and GIG-ICA in experiment 1

**Figure 3:**
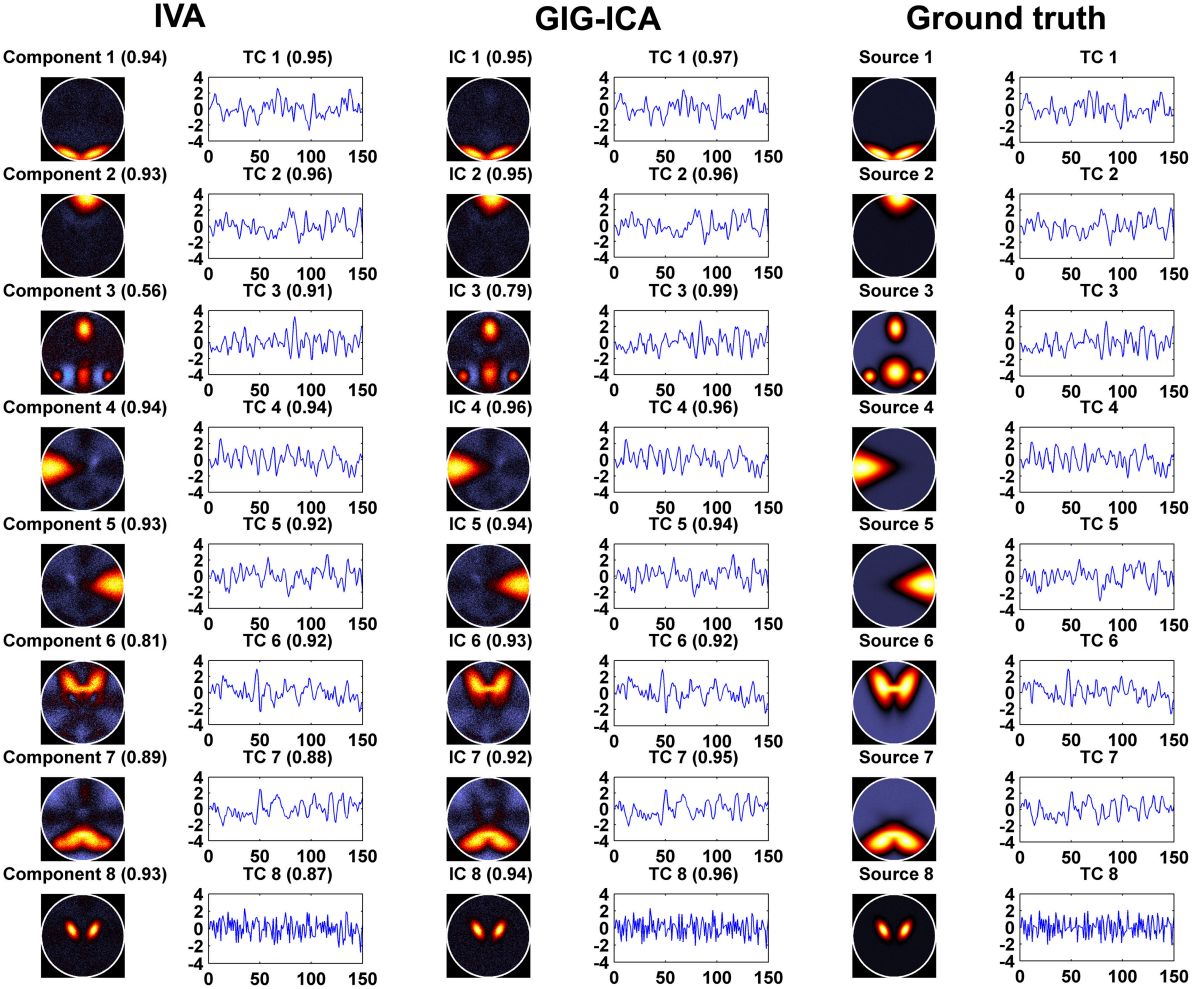
**The estimated components/TCs of one subject obtained from IVA and GIG-ICA when the CNR = 1**. The value in parenthesis close to each estimated component/TC is the relevant correlation coefficient between the component/TC and the simulated ground truth (GT) source/TC. The GT sources/TCs are also shown for comparisons.

**Figure 4:**
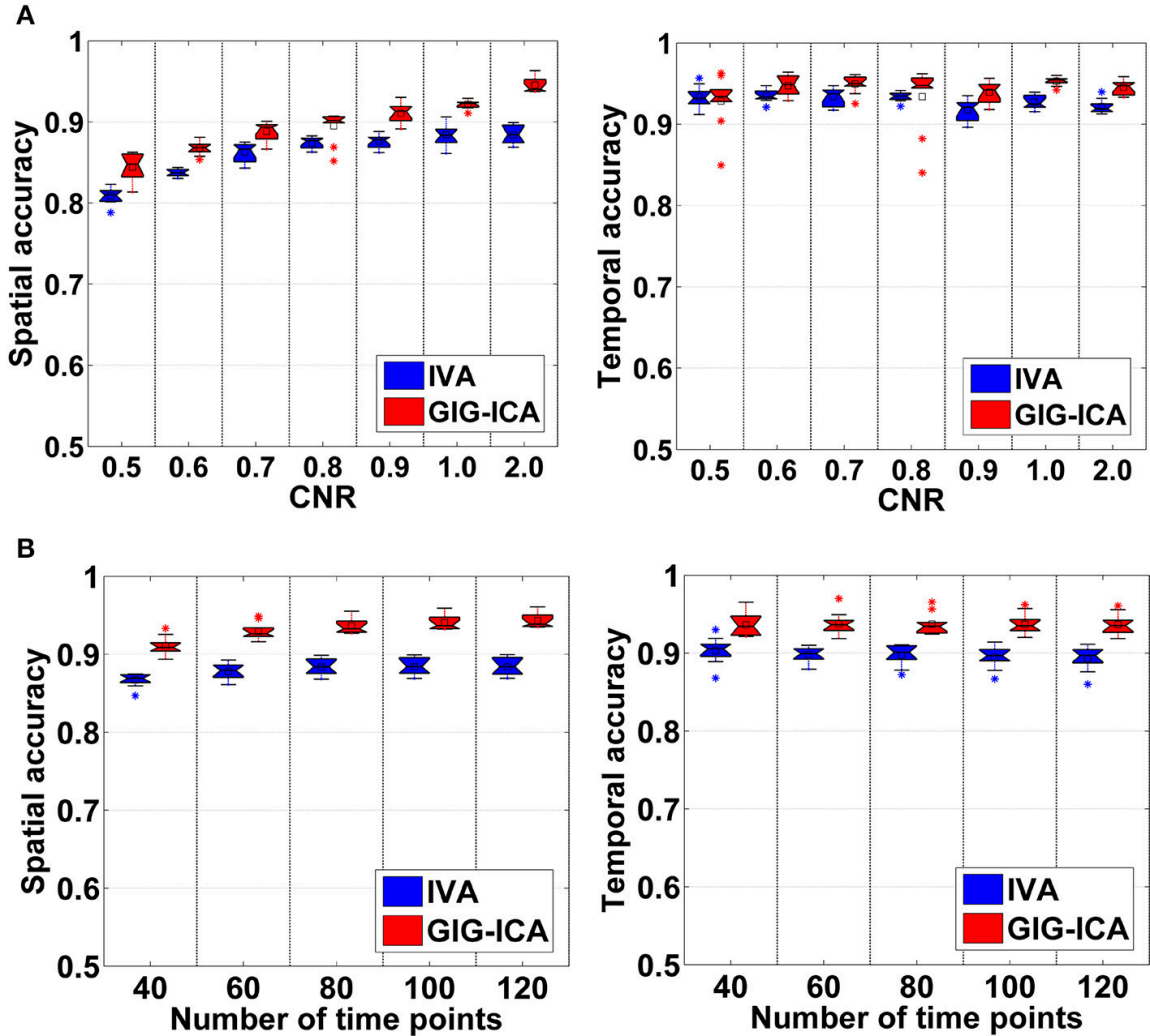
**(A)** Spatial and temporal accuracy measure obtained from IVA and GIG-ICA under different CNRs ranging from 0.5 to 2. **(B)** Spatial and temporal accuracy measure obtained from IVA and GIG-ICA under different numbers of time points. The x-axis in each boxplot denotes CNR in **(A)** or number of time points in **(B)**. The y-axis denotes the mean of spatial/temporal correlation coefficients between one subject's estimated components/TCs and the corresponding ground truth sources/TCs, which was used to measure the overall spatial/temporal accuracy of one subject's result. Each point in a given boxplot corresponds to the overall spatial/temporal accuracy of one subject. For each boxplot, the central line is the median, and the edges of the box are the 25 and 75th percentiles. The whiskers extend to 1 inter-quartile range, and each outlier is displayed with a “^*^” sign. The mean value is indicated by a square. Subsequent boxplots are formatted similarly.

Figure 3 shows the components/TCs of one subject estimated by IVA and GIG-ICA from the simulated data with the CNR = 1. For this case, we can see that both methods can generally recover all of spatial components and TCs. For some components (e.g., component 3, 6, and 7), GIG-ICA had slightly higher component/TC accuracy than IVA. Figure 4A summarizes the comparison results across 10 subjects under the condition of different CNRs. It can be observed that the recovery accuracy of components by both methods was improved along with the increasing of CNR while TCs recovery was relatively insensitive to different CNRs. Measured by the mean accuracy, GIG-ICA outperformed IVA across most of CNR settings in terms of component/TC accuracy. Figure 4B demonstrates results from evaluating the influence of different numbers of time points. Both methods showed increasing recovery accuracy of components with more time points used. Paired *t*-test results (Table 2) show that for most of the CNR and time point settings tested, GIG-ICA showed significantly increased accuracy (especially the spatial accuracy) than IVA. Our results indicate the advantage of GIG-ICA in recovering subject-common sources than IVA, and GIG-ICA can yield components with higher accuracy even under the case of low quality and quantity of data.

**Table 2:** **Results of the estimation accuracy using paired ***t***-tests for Experiment 1**.

**Data with different quality**	**CNR = 0.5**	**CNR = 0.6**	**CNR = 0.7**	**CNR = 0.8**	**CNR = 0.9**	**CNR = 1.0**	**CNR = 2**
*p*-value in spatial accuracy	5.43e-05	1.16e-06	3.05e-06	7.96e-03	1.42e-07	2.79e-06	1.77e-07
*t*-value in spatial accuracy	−7.14	−11.44	−10.19	−3.39	−14.61	−10.30	−14.24
*p*-value in temporal accuracy	0.61	0.002	4.34e-05	0.98	3.12e-05	6.20e-06	7.14e-06
*t*-value in temporal accuracy	0.52	−4.12	−7.35	0.014	−7.66	−9.36	−9.20
**Data with different quantity**	**Number of time points** = **40**	**Number of time points** = **60**	**Number of time points** = **80**	**Number of time points** = **100**	**Number of time points** = **120**		
*p*-value in spatial accuracy	2.21e-05	2.01e-06	9.17e-07	4.71e-07	2.98e-07		
*t*-value in spatial accuracy	−8.00	−10.71	−11.76	−12.71	−13.41		
*p*-value in temporal accuracy	3.74e-03	2.0e-3	1.58e-3	8.52e-05	5.64e-05		
*t*-value in temporal accuracy	−3.88	−6.01	−6.21	−6.73	−7.10		

#### Component and time course accuracy estimated from IVA and GIG-ICA in experiment 2

**Figure 5:**
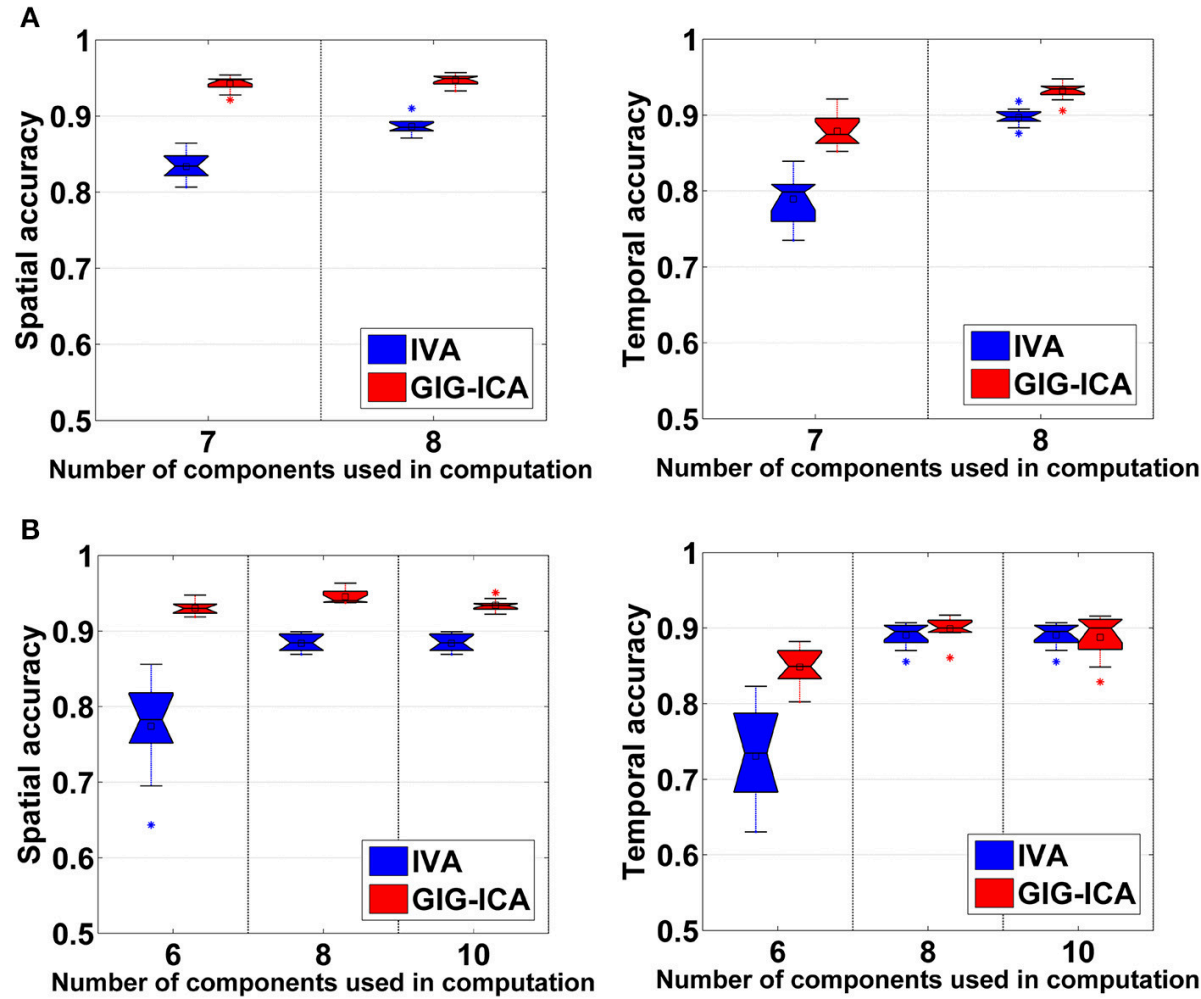
**(A)** Spatial and temporal accuracy obtained from IVA and GIG-ICA under different model orders for datasets with varied numbers of sources across subjects. **(B)** Spatial and temporal accuracy obtained from IVA and GIG-ICA under the model order as 6, 8, and 10 for datasets generated with 8 sources. The x-axis in each boxplot denotes the number of components used in computation. The y-axis denotes the mean of spatial/temporal correlation coefficients between one subject's estimated components/TCs and the corresponding ground truth sources/TCs, which was used to measure the overall spatial/temporal accuracy of one subject's components/TCs.

Figure 5A shows the accuracy results obtained from data with varied numbers of sources across different subjects. We can see that under all model orders (i.e., different numbers of components), GIG-ICA showed significantly better performance (see Table 3) than IVA, indicating that GIG-ICA is able to tolerate source number variation and is also not very sensitive to the number of components used.

**Table 3:** **Results of the estimation accuracy using paired ***t***-tests for Experiment 2**.

	**Data with varied source number**		**Inaccurate number of component**		
	**IC number = 7**	**IC number = 8**	**IC number = 6**	**IC number = 8**	**IC number = 10**
*p*-value in spatial accuracy	1.32e-09	2.47e-09	2.24e-05	1.76e-07	1.33e-07
*t*-value in spatial accuracy	−24.86	−23.18	−7.99	−14.25	−14.72
*p*-value in temporal accuracy	8.10e-06	7.25e-05	4.10e-04	0.10	0.77
*t*-value in temporal accuracy	−9.06	−6.88	−5.44	−1.85	0.30

The results demonstrated in Figure 5B were obtained using data generated with 8 sources and different numbers of components for computation (i.e., 6, 8, and 10). It can be observed that GIG-ICA significantly outperformed IVA under all model orders in terms of the spatial accuracy (see Table 3). Regarding the temporal accuracy, GIG-ICA had significantly greater accuracy using the model order 6, but slightly decreased accuracy using the model order 10, compared to IVA. For the model order 8, the TC results of the two methods are statically close. When the used number of components was the same as the real source number (i.e., 8), both methods achieved the best estimation. When the number of components was 10, there was a slight decrease in recovering components/TCs for both methods, compared to the results of the model order as 8. However, when the number of sources was underestimated (i.e., 6), there was a significant drop of accuracy for the IVA but a slight decrease for GIG-ICA. Because the accurate number of components is very difficult to estimate correctly in practice, the relative insensitivity of GIG-ICA to the model order may provide an important benefit.

#### Component and time course accuracy estimated from IVA and GIG-ICA in experiment 3

**Figure 6:**
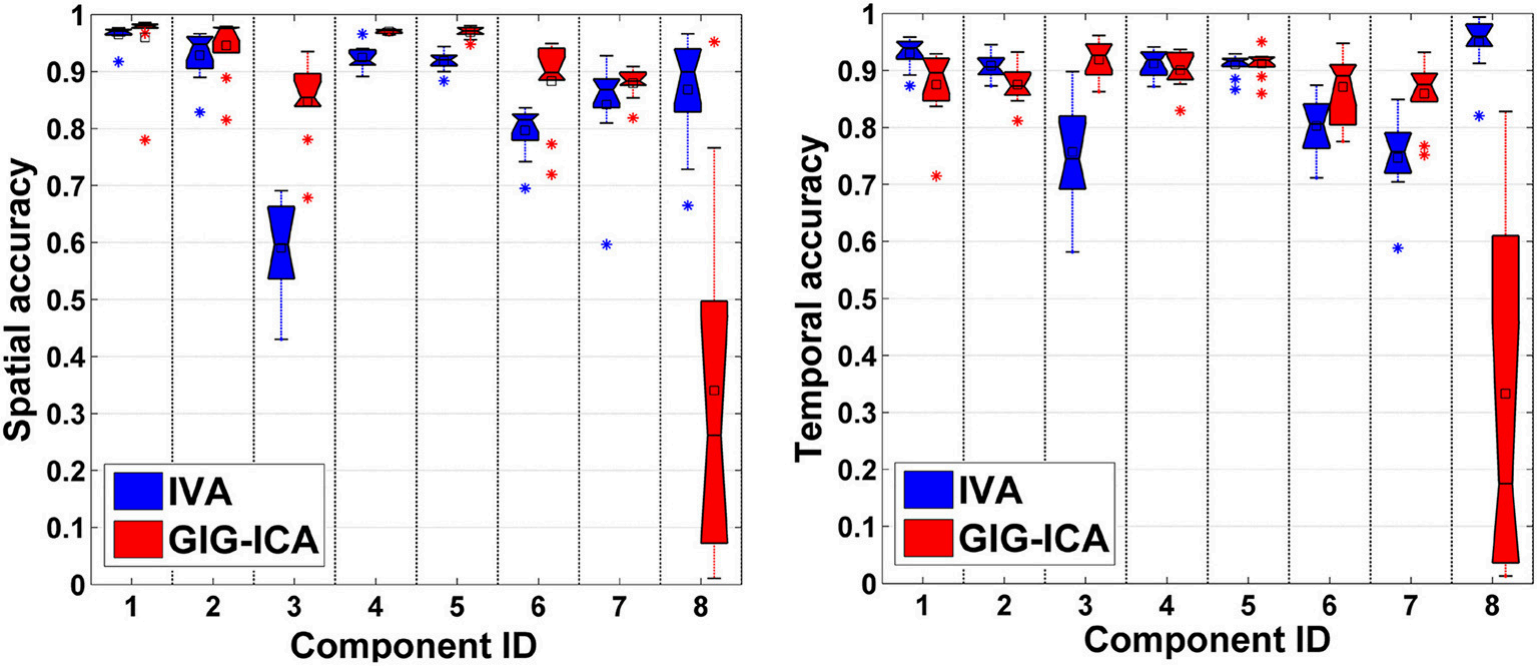
**Spatial and temporal accuracy of each estimated component and TC obtained from IVA and GIG-ICA for datasets with subject-unique sources**. The 8th component is subject-unique. The y-axis denotes the spatial/temporal correlation between one subject-specific component/TC and the corresponding ground truth source/TC. The accuracy metrics of each component/TC from all subjects are shown using one boxplot. Each point in one boxplot corresponds to the spatial/temporal accuracy of one component/TC for one subject.

In this experiment, we tested the two methods using datasets where each subject had a spatially unique source. Accuracy of each estimated individual component/TC is shown in Figure 6. It is seen that for the estimated spatial components, measured by the mean accuracy across subjects, GIG-ICA had a better performance for the subject-common sources (i.e., the first 7 sources), but showed a worse estimation for the subject-unique source (i.e., the 8th source) than IVA. Regarding the estimated eight TCs, measured by the mean accuracy across subjects, GIG-ICA had higher TC accuracy in four TCs and decreased TC accuracy in terms of the subject-unique source compared to IVA. Using paired *t*-tests (see Table 4), among the 7 subject-common sources, four components and three TCs were significantly more accurate using GIG-ICA than using IVA. IVA outperformed GIG-ICA in estimating the subject-unique sources (passing *p* < 0.05 with correction). Our results suggest that for the data generated with subject-unique sources, in general GIG-ICA still performed well for the similar sources but did not work well for the unique source. In contrast, IVA can estimate the subject-unique source and its associated TC with high accuracy.

**Table 4:** **Results of the estimation accuracy using paired ***t***-tests for Experiment 3**.

	**Component 1**	**Component 2**	**Component 3**	**Component 4**	**Component 5**	**Component 6**	**Component 7**	**Component 8**
*p*-value in spatial accuracy	0.70	0.02	4.34e-08	1.04e-04	2.01e-07	1.01e-03	0.21	8.95e-04
*t*-value in spatial accuracy	0.39	−2.66	−16.74	−6.56	−14.04	−4.78	−1.33	4.86
*p*-value in temporal accuracy	0.03	0.03	3.81e-04	0.20	0.78	5.53e-03	3.29e-05	1.96e-04
*t*-value in temporal accuracy	2.53	2.43	−5.50	1.38	−0.29	−3.62	−7.61	6.03

### Results from test-retest resting-state fMRI data

**Figure 7:**
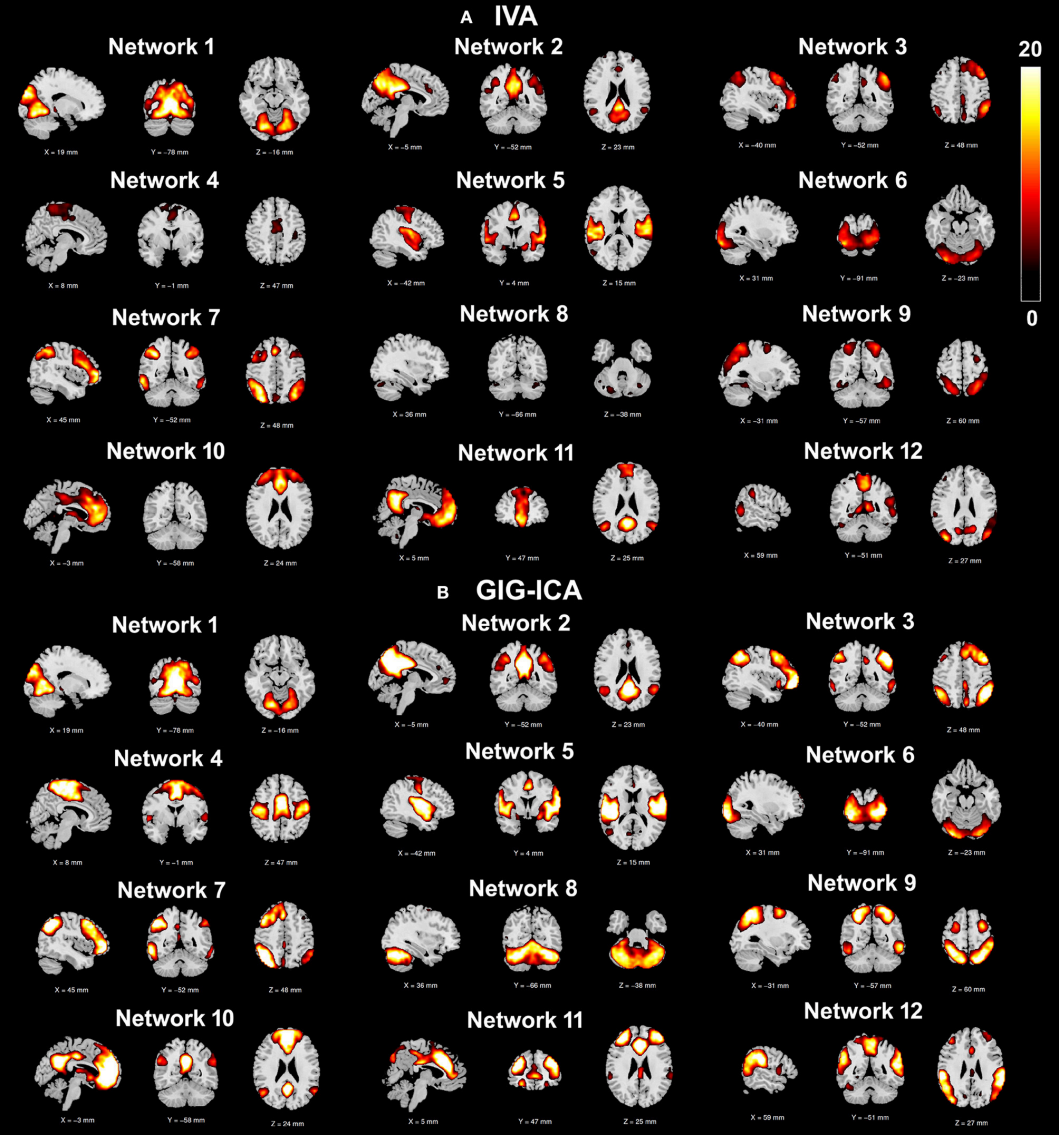
**One-sample ***t***-test ***t***-value maps (***p*** < 0.01 with FDR correction) of the 12 matched networks, obtained by (A)** IVA and **(B)** GIG-ICA under the case of the model order as 30. The 12 matched networks shown are sorted according to the similarity (i.e., correlation) between networks from the two methods.

**Figure 8:**
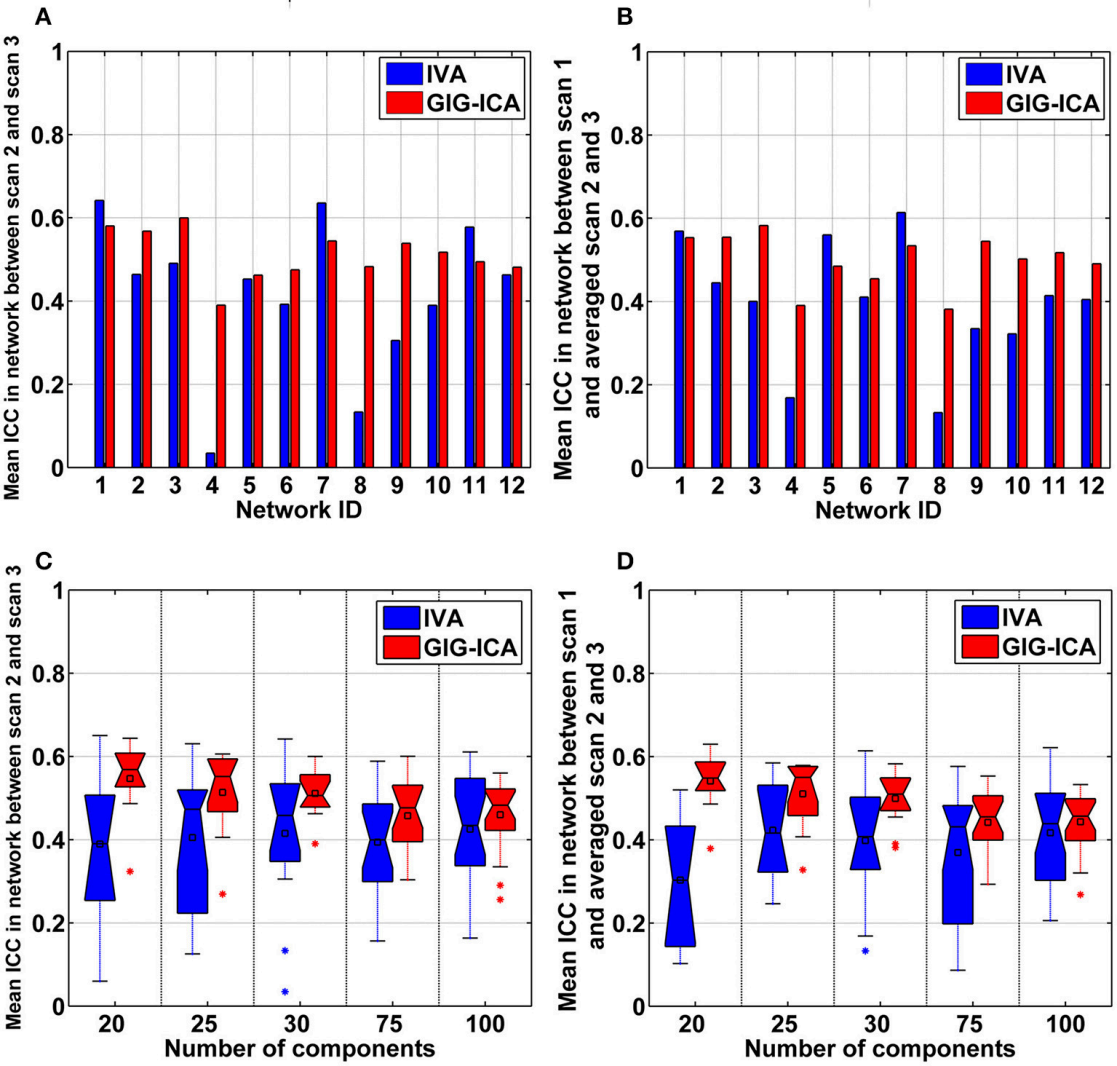
**(A,B)** Reliability measures of the 12 selected networks for IVA and GIG-ICA under the case of the model order as 30. The x-axis denotes the network ID which corresponds to that in Figure 7. **(A)** Mean ICC value in each network reflecting the short-term reliability of the network. The value was obtained by first computing ICCs between the corresponding networks of scan 2 and that of scan 3, and then averaging ICCs in the significant voxels. **(B)** Mean ICC value in each network reflecting the long-term reliability of the network. The value was obtained by first computing ICCs between the corresponding networks from scan 1 and the mean networks of scan 2 and 3, and then averaging ICCs in the significant voxels. **(C,D)** The summarized network reliability measures for IVA and GIG-ICA under different model orders (i.e., different numbers of components). **(C)** Short-term reliability of networks. **(D)** Long-term reliability of networks. Each boxplot shows the reliability measures of different networks using IVA or GIG-ICA with one given model order. For the model order 20, 25, 30, 75, and 100, the number of matched networks between the two methods were 9, 10, 12, 19, and 22, respectively.

Using the test-retest resting-state fMRI datasets, we assessed the individual-level spatial networks in terms of their short-term and long-term reliability. Figure 7 shows the one-sample *t*-tests results of the 12 matched networks for the two methods under the condition of the model order as 30. We found that compared to IVA, GIG-ICA in general showed higher *t*-values for all networks. For the case of the model order as 30, the short-term and long-term reliability measures of each network are demonstrated in Figures 8A,B, respectively. Results indicate that for most of the networks, greater reliability measures were obtained using GIG-ICA compared to IVA, although there were also four networks (including Network 1, Network 5, Network 7, and Network 11) showing slightly higher short-term or long-term reliability in IVA than GIG-ICA. Furthermore, some networks including the sensorimotor and cerebellum-related networks from IVA had very low reliability. To summarize, we show the short-term and long-term reliability of all networks estimated with different model orders in Figures 8C,D. It can be seen that measured by the mean values of reliability measures across all networks, the higher network reliability was achieved by GIG-ICA than IVA for all model order settings.

**Figure 9:**
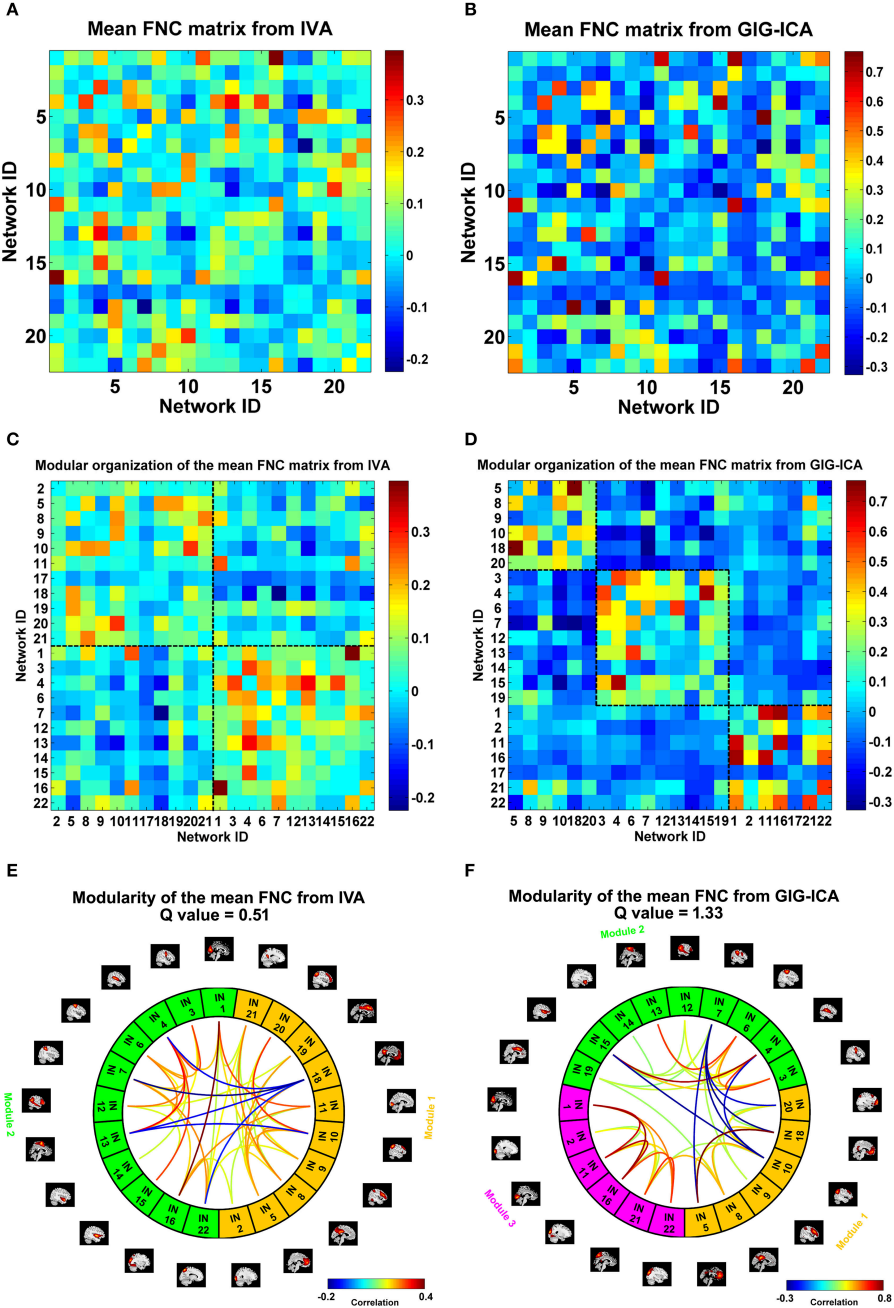
**The mean FNC matrix and its modularity result with the model order as 100. (A,B)** The mean FNC matrix across subjects derived from IVA and GIG-ICA. There were 22 matched networks between the two methods. **(C,D)** Modular organization of the mean FNC matrix from IVA and GIG-ICA. **(E,F)** The connectogram representation of the modularity of the mean FNC obtained from IVA and GIG-ICA. In **(E,F)**, the intrinsic networks (INs) belonging to the same modular are shown using the same color, and only top 20% of the connectivities with higher absolute connectivity strengths among networks are shown using lines.

We also compared the two methods in constructing functional interaction among networks. Under a model order of 100, 22 networks were highly matched between the two methods. For each dataset, one FNC matrix was generated based on the associated TCs of the 22 networks. Figures 9A,B show the mean FNC matrix across all 75 datasets for IVA and GIG-ICA, respectively. It is observed that the two FNC matrices generally showed a similar pattern. However, the contrast in FNC appeared higher in GIG-ICA than IVA. According to the modularity segmentation of networks, we reorganized the mean FNC matrix's structure for IVA (Figure 9C) and GIG-ICA (Figure 9D). The identified modules for the two methods were demonstrated in Figures 9E,F, respectively. Three modules mainly relating to the default mode network (module 1), the cognitive control, sensorimotor and auditory functions (module 2), and the vision function (module 3) were found using GIG-ICA. Module 1 and 2 showed anti-correlations in their connectivities. Regarding IVA, two modules were detected, while the vision-associated networks were separated into two modules. Furthermore, the modularity quality was greater in GIG-ICA (*Q* = 1.33) compared to IVA (*Q* = 0.51) when the number of components was 100.

**Figure 10:**
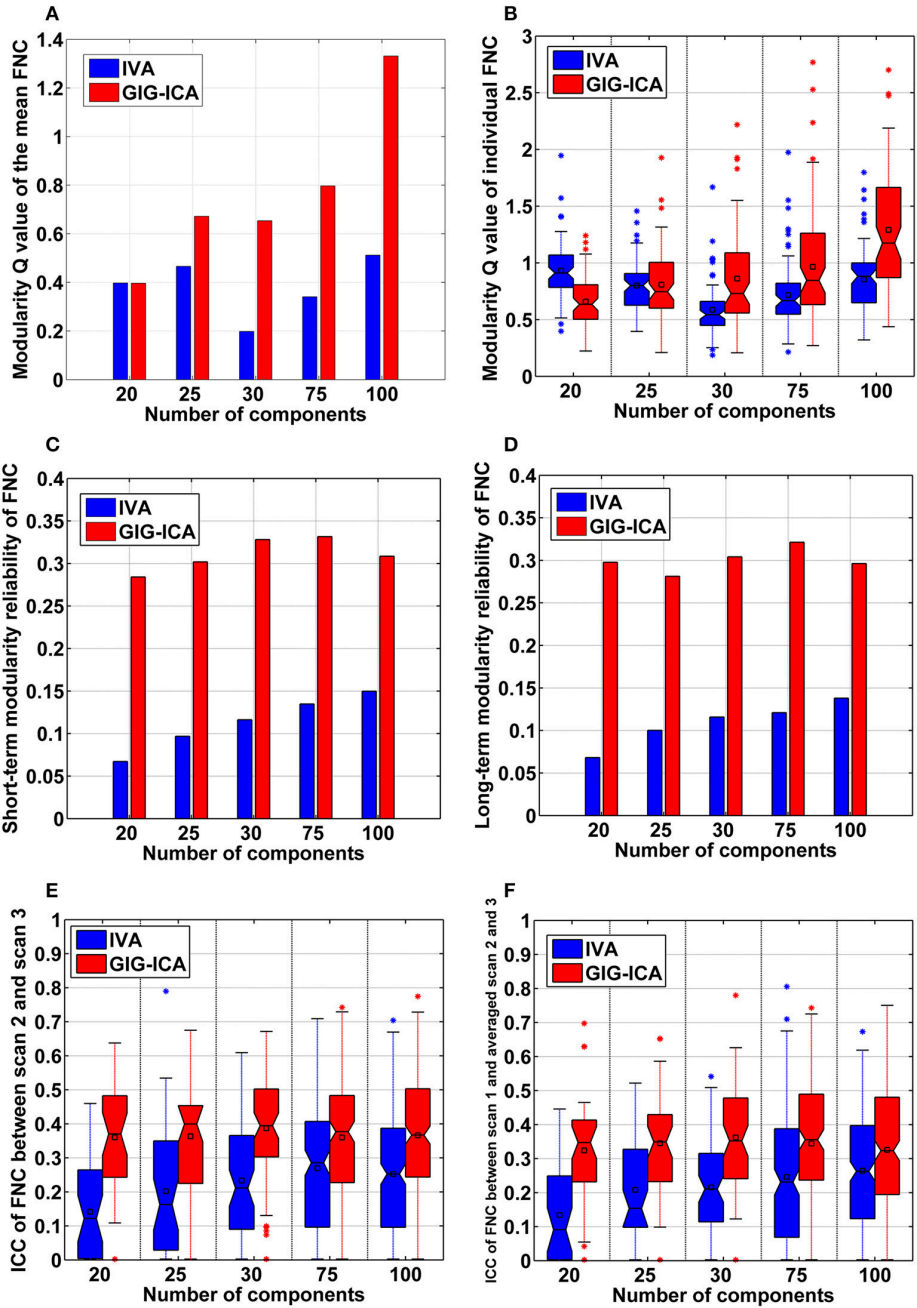
**The summarized modularity and reliability measures of FNC for IVA and GIG-ICA under different model orders (i.e., different numbers of components). (A)** The modularity *Q*-value of the mean FNC. **(B)** Individual FNC's modularity *Q*-value. The *Q*-values of FNC matrices from all datasets are shown using one boxplot, each point of which corresponds to a *Q*-value of one individual FNC matrix. **(C)** The short-term modularity reliability. **(D)** The long-term modularity reliability. **(E)** The short-term reliability of connectivity strengths in FNC. **(F)** The long-term reliability of connectivity strengths in FNC. In **(E,F)**, each boxplot includes ICC values of all connectivities.

Furthermore, GIG-ICA showed an equivalent or higher modularity *Q*-value of the mean FNC than IVA for the model order settings tested (see Figure 10A). Regarding individual FNC's modularity, Figure 10B demonstrates that excepting the low model order 20, GIG-ICA with a greater mean *Q*-value outperformed IVA for most of the cases. Moreover, measured by the AMI, both the short-term and the long-term modularity reliability metrics were greater in GIG-ICA than IVA for all tests, as shown in Figures 10C,D. Both the short-term and long-term ICC measures (Figures 10E,F) support that the connectivity strengths in FNC were generally more robust using GIG-ICA method, compared to IVA.

**Figure 11:**
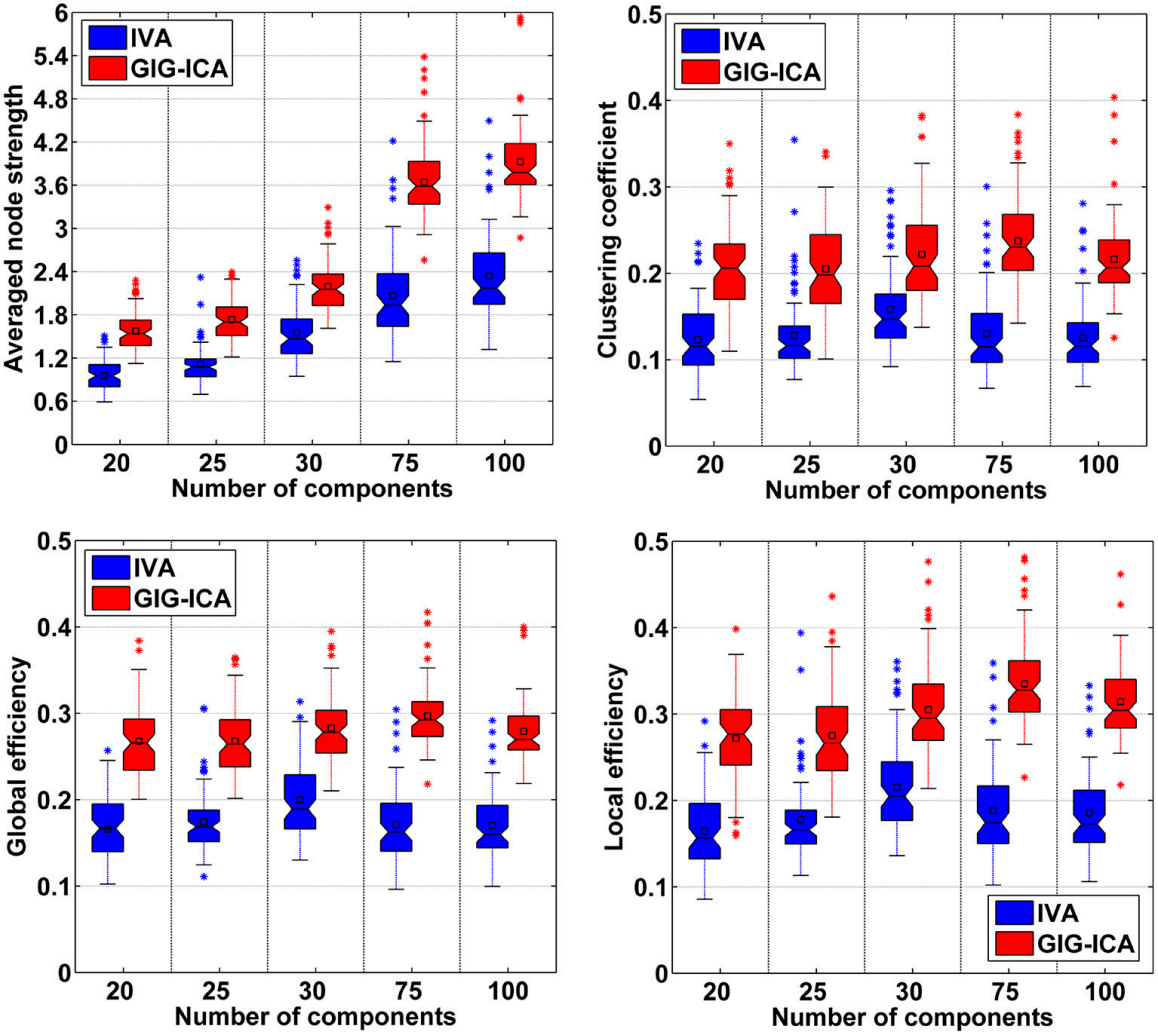
**Comparison of graph theory based metrics of FNC under different model orders**. In the left-top subfigure, the averaged node strength values computed based on all 75 datasets' FNC matrices from IVA/GIG-ICA with one specific model order are shown in one boxplot. Other boxplots are formatted similarly.

As mentioned in the method section, we also examined other graph-theory based metrics for individual FNC. The summarized results for the averaged node strength, clustering coefficient, global efficiency, and local efficiency are shown in (Figures 11), suggesting that GIG-ICA resulted in higher mean values in all these graph metrics than IVA under all model order settings.

## Discussion

In this work, we compared two promising approaches (i.e., IVA and GIG-ICA) for analyzing multi-subject fMRI data. Both methods can estimate subject-specific brain functional networks with correspondence across different subjects. IVA considers both the independence of individual components and the dependence of similar components across subjects. GIG-ICA first estimates the group-level ICs from all data and then computes the subject-specific ICs with the group-level ICs as guidance. Using simulations, we investigated if the two methods can yield accurate individual-level components and time courses under different conditions, including different data quality (i.e., CNR) and data quantity (i.e., number of time points), varied number of sources and inaccurate number of components, as well as presence of spatially subject-unique sources. Furthermore, we assessed their performance using test-retest resting-state fMRI data with respect to spatial networks' reliability and graph-theory based metrics of FNC under different model orders.

In Experiment 1 using simulations, we evaluated the two methods using data with various quality and quantity. Our results suggest that both IVA and GIG-ICA showed improved performance along with the increased CNR and time points of data. For the sources with slight inter-subject spatial variability, GIG-ICA obtained components with higher accuracy than IVA, and performed very well under the case of low CNR and less time points. It is known that both IVA and GIG-ICA require a fixed number of components for computation, generating the same number of components for all subjects. When datasets of different subjects are simulated using different numbers of sources, the resulting components of some subjects have different numbers with the real number of sources. So, in Experiment 2, we simulated varied number of sources between different groups and also investigated the influence of inaccurate number of components. Our results suggest that GIG-ICA showed a relatively better performance and was stable to the various numbers of sources under this case. We also tested the two methods in terms of the effect of the number of components, indicating that IVA gave rise to a significant reduced accuracy when the model order was underestimated while GIG-ICA was not very sensitive to the inaccurate model order.

All the above mentioned experiments were applied to the datasets generated using sources that were similar across subjects. In Experiment 3, using datasets where all subjects had a subject-unique source with large inter-subject spatial variability, we found that IVA significantly showed a better performance in the component/TC accuracy of the unique source than GIG-ICA, although GIG-ICA in general still performed better for other subject-common sources compared to IVA. This is likely due to that the two methods are different in algorithm level. GIG-ICA first extracts the group-level components, and then estimates the corresponding individual-level components for each individual-subject's data. In contrast, IVA simultaneously estimates the individual-subject's components and optimizes the dependence of components across different subjects. Therefore, we suggest using GIG-ICA to estimate networks that are consistent across subjects, while IVA is more appropriate for networks with significant inter-subject variability. IVA's superiority in estimating subject-unique sources possibly enables it to be more suitable to data from patients with particular brain structure damage, such as patients suffering from brain tumor that could result in greatly different functional networks. Our previous work (Du et al., 2014, 2015c, 2017) showed that GIG-ICA performed well for fMRI data from healthy controls and patients with mental disorders, which are supposed to have similar network patterns but subtle differences. In fact, all GICA approaches (Calhoun et al., 2001, 2009; Beckmann et al., 2009; Erhardt et al., 2011) have the same limitation with GIG-ICA, since all of them back-reconstruct individual-subject's ICs based on the group-level ICs. However, as our previous work (Du et al., 2016a) suggested, GIG-ICA is a powerful approach for main fMRI researches, due to the fact that the subject-common networks can be estimated and denoised without having to accurately estimate the artifacts. In future, a general framework that leverages the strengths of IVA and GIG-ICA is expected for achieving high accuracy of both subject-common and subject-unique networks.

Our experiments using healthy participants' test-retest resting-state fMRI data revealed that regardless of low model order and high model order, GIG-ICA in general obtained functional networks with relatively greater short-term and long-term reliability compared to IVA, although a few networks showed slightly higher reliability in IVA than GIG-ICA. In terms of the interaction among networks represented by FNC, we found that the mean FNC matrix from the two methods showed a similar pattern to some extent. However, both the mean FNC and the individual-level FNC showed stronger modularity (i.e., *Q*-value) using GIG-ICA compared to IVA for most of the model order settings examined. Measured by the AMI, the modular structure was more reliable during short-term and long-term rescanning using GIG-ICA for all tests, compared to using IVA. Despite short-term and long-term, ICC measures demonstrate that connectivity strengths were generally more robust using GIG-ICA method, compared to IVA. Moreover, FNC obtained from GIG-ICA showed relatively higher values in the averaged node strength, clustering coefficient, global efficiency, and local efficiency, indicating stronger interaction among brain functional networks.

There are some limitations in our work. (1) The simulations are quite simple. Only eight sources and ten subjects were simulated, while the proportion in fMRI data is certainly greater. In practical applications, there exist more complex situations that could involve many subject-unique sources, high diversity in source number, and great bias in model order estimation. Therefore, it's possible that conclusions we draw from simulations are over-simplified and of limited applicability. However, we also evaluated the two methods using data with more subject-common and subject-unique sources. The results are included in the Supplementary Materials (Figures S2, S4). Our results suggest that the performances of both methods were affected by greater spatial overlapping among sources, and the presence of more subject-unique sources may slightly influence the estimations of the subject-common sources in GIG-ICA to some extent. (2) The number of sources in real data is difficult to estimate accurately. Therefore, we don't know the appropriate model orders at which to compare these two methods in real data. We compared the two methods using different numbers of components and found similar results, but these methods may yield different performances with other model orders. (3) Since IVA involves a more complicated optimization task, performance might improve if a best run selection mechanism as in previous work (Ma et al., 2011) is used to select the most reliable run across multiple runs. However, we did not perform estimation of multiple runs due to the computation load that would significantly increase the computation time. Similarly use of a more powerful IVA algorithm such as the one proposed in Boukouvalas et al. (2015) might improve the estimation performance at the expense of computation cost. (4) Using healthy participants' test-retest resting-state fMRI data, GIG-ICA obtained higher network reliability as well as stronger and more reliable modularity than IVA. Network reliability is regarded as a desirable property since the fMRI data in our experiments were from healthy subjects' test-retest scans (Shehzad et al., 2009; Zuo and Xing, 2014). Previous researches (Wang et al., 2010; Bullmore and Bassett, 2011) have supported that healthy brain's intrinsic activity is organized as a small-world, highly efficient network with highly connected brain regions. Nevertheless, the truths regarding both network reliability and integration are unknown for real data. In the future, we will employ fMRI data from both healthy controls and patients with mental disorders to examine the ability of the two methods in identifying potential biomarkers.

## Ethics statement

The fMRI data we used are open. These data are fully available via http://www.nitrc.org/projects/nyu_trt. Data were collected according to protocols approved by the institutional review boards of New York University (NYU) and the NYU School of Medicine. The related information can be found in Zuo et al. (2010). Reliable intrinsic connectivity networks: test-retest evaluation using ICA and dual regression approach.

## Author contributions

YD designed the study; analyzed and interpreted the data; revised the manuscript; and gave final approval. DL interpreted the results; drafted the manuscript and gave final approval. QY interpreted the results; revised the manuscript and gave final approval. JS interpreted the data; revised the manuscript and gave final approval. JC interpreted the results; revised the manuscript and gave final approval. SR helped with IVA implementation, interpreted the data; drafted and revised the manuscript; gave final approval. TA interpreted the data; drafted and revised the manuscript; gave final approval. VC designed the study; interpreted the data; drafted and revised the manuscript; gave final approval.

### Conflict of interest statement

The authors declare that the research was conducted in the absence of any commercial or financial relationships that could be construed as a potential conflict of interest.
