# Supplementary material for: Comparison of IVA and GIG-ICA in Brain Functional Network Estimation Using fMRI Data
Source: Front Neurosci. 2017 May 19;11:267. doi: 10.3389/fnins.2017.00267 (PMC5437155; doi:10.3389/fnins.2017.00267)
Supplement: Supplementary file 1 [file DataSheet1.DOCX]

**Supplementary materials**

**Method of matching two groups of components**

In order to make two groups of components to be comparable, we matched them using a greedy rule. Firstly, one similarity matrix $\mathbf{C}$ (size: $a\times b$) between $a$ components from the first group and $b$ components from the second group was initially obtained by computing the absolute values of their Pearson correlation coefficients. Based on the matrix $\mathbf{C}$, two components (e.g., component $a_{i}$ and component $b_{j}$) from the two groups relating to the highest correlation value were selected and taken as the first-matched two components. And then, all correlation values associated with the first-matched two components in the matrix $\mathbf{C}$ were set to zero, resulting in a new correlation matrix $\mathbf{C}_{\mathbf{new}}$. Similarly, the second-matched two components can be identified based on the new correlation matrix $\mathbf{C}_{\mathbf{new}}$ using the same manner. Finally, all components were matched across the two groups.

## Supplementary simulation-based experiments

*Supplementary Experiment 1: Comparing IVA and GIG-ICA using data with more sources*

In the main manuscript, considering few pixels in the simulated 2D images (148×148 pixels), we used 8 spatial templates in SimTB toolbox to yield the independent sources for each subject and the relatively great spatial variability of sources across different subjects. The ratio of the number of pixels in mask to the number of sources (i.e., 8) was greater than 2000, which is consistent with the real case of nearly 60,000 voxels in brain mask and 20-30 components usually used. In this experiment, we simulated more sources (i.e., 16 sources) to evaluate the methods. Fig. S1 shows the simulated 16 sources and time courses of two subjects as well as the spatial variability of sources across different subjects. Spatial variability was simulated using the same parameters with that in Experiment 1. For each subject, we simulated 4 datasets with different CNRs (including 0.5, 1, 1.5 and 2) and a fixed number of time points (i.e., 150). For each subject, we also had 5 datasets with the time points from 40 to 120 in steps of 20 while the CNR was fixed at 2. Similar to the procedure in Experiment 1, we then compared IVA and GIG-ICA using data with different CNRs and different time points. The number of components (i.e., $G1$, $G2$, $I1$) was set to the number of true sources (i.e., 16).

Fig. S2 (A) and (B) summarize the comparison results under the conditions of different CNRs and different time points, respectively. It can be observed that the estimation accuracy in general showed an increased trend along with better data quality and greater data quantity for both methods. GIG-ICA significantly outperformed IVA in terms of the source estimation for all tests. In some cases (i.e., the cases of lower CNRs and less time points), GIG-ICA had decreased accuracy in the obtained time courses possibly due to the great overlapping of multiple sources in simulations and limited information to use. Compared to the results in Experiment 1 which included less sources, the spatial and temporal accuracy from both methods were lower in this experiment, likely resulting from greater spatial overlapping.

**
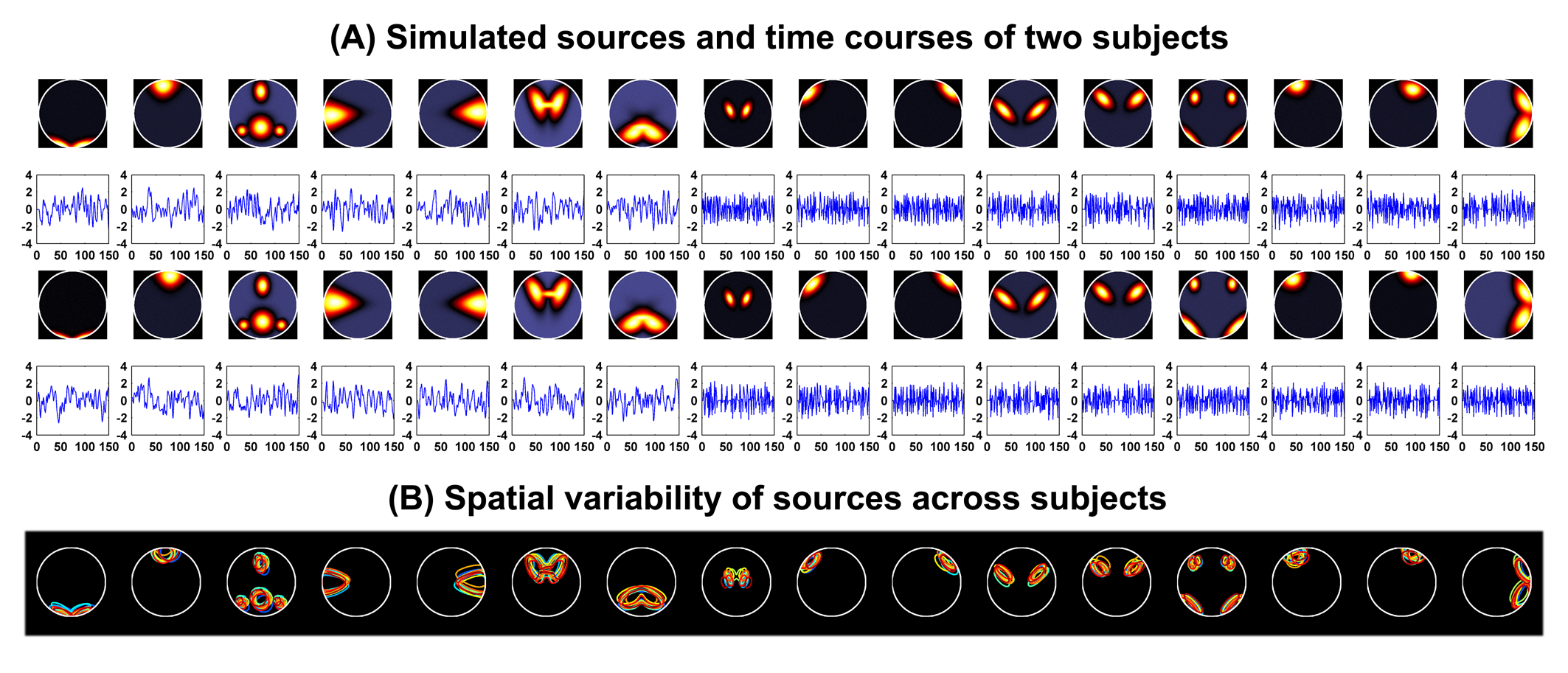
**

Fig. S1. (A) The simulated 16 sources and their associated time courses of two subjects in supplementary Experiment 1. (B) The spatial variability of sources across subjects in supplementary Experiment 1. Each color denotes the source contours of a different subject.


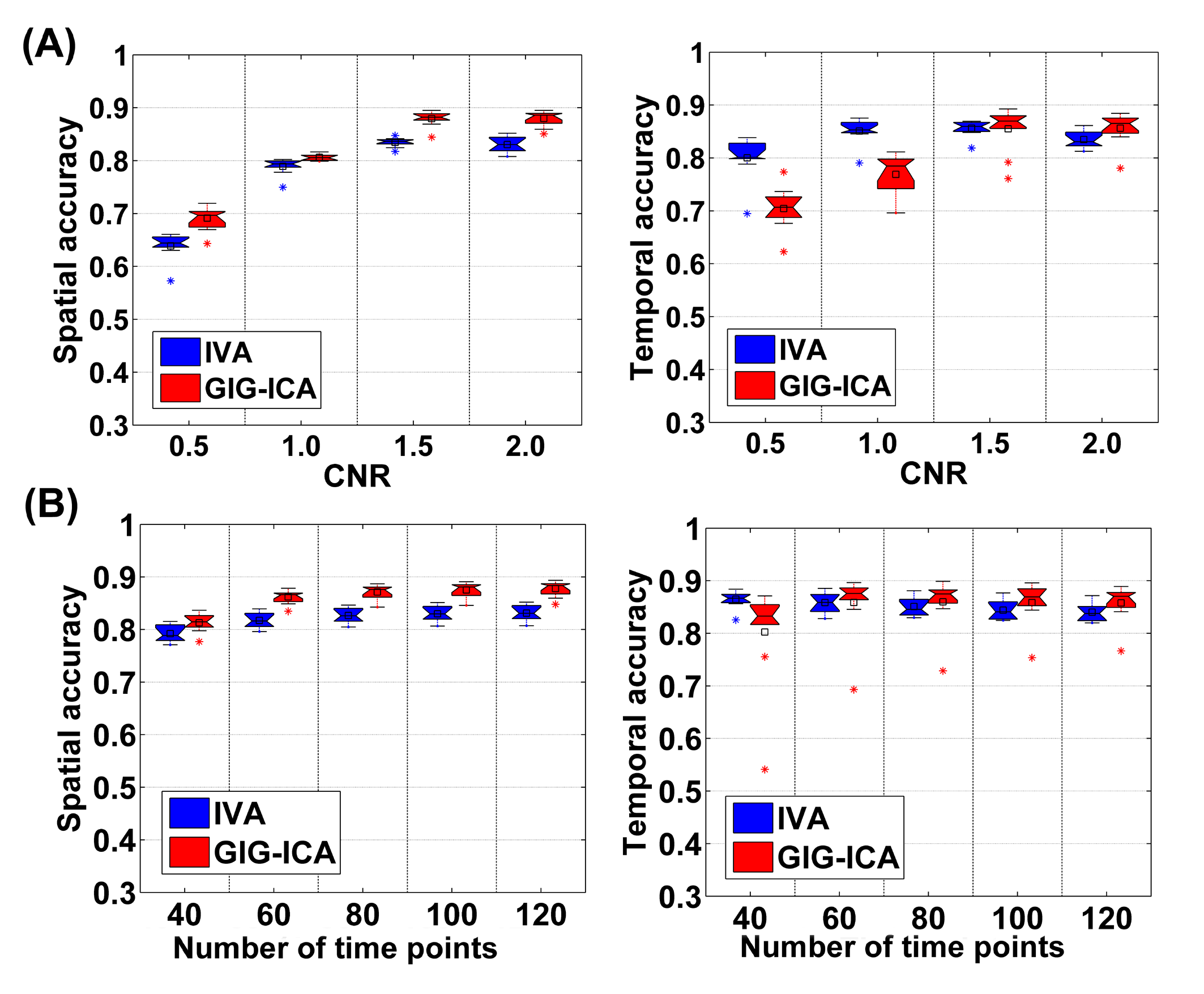


Fig. S2. Results of supplementary Experiment 1. (A) Spatial and temporal accuracy measure obtained from IVA and GIG-ICA under different CNRs including 0.5, 1, 1.5 and 2. (B) Spatial and temporal accuracy measure obtained from IVA and GIG-ICA under different numbers of time points. The x-axis in each boxplot denotes CNR in (A) or number of time points in (B). The y-axis denotes the mean of spatial/temporal correlation coefficients between one subject’s estimated components/TCs and the corresponding ground truth sources/TCs, which was used to measure the overall spatial/temporal accuracy of one subject’s result. Each point in a given boxplot corresponds to the overall spatial/temporal accuracy of one subject.

*Supplementary Experiment 2: Comparing IVA and GIG-ICA using data with more subject-unique sources*

In Experiment 3, we simulated one subject-unique source for each dataset and compared the two methods. In this experiment, we assessed using data with more subject-unique sources. Each subject’s data was simulated using 7 subject-common sources with moderate variability and two subject-unique sources. The 7 subject-common sources were generated using the same templates and parameters (i.e., translations, rotations, spreads) with that in Experiment 3. The additional two sources (i.e., the 8^th^ and 9^th^ sources) showed unique patterns and were greatly different in all subjects. Fig. S3 shows the two subject-unique sources and their related TCs of all subjects. The number of components was specified as the real number of sources (i.e., 9) for computation.

Fig. S4 shows the accuracy of each estimated individual component/TC. Compared to IVA, GIG-ICA in general had a better or similar performance for the subject-common sources in the spatial accuracy. For the two subject-unique sources, IVA significantly outperformed GIG-ICA in component/TC accuracy. Some associated TCs of the subject-common sources were more accurately estimated by IVA. Compared to the results of Experiment 3 in the main manuscript, it seems like that the estimation accuracy of the subject-common sources had slight reduction in GIG-ICA. We suspect that the estimation of subject-common sources could be affected by the presence of abundant subject-unique sources in GIG-ICA.

**
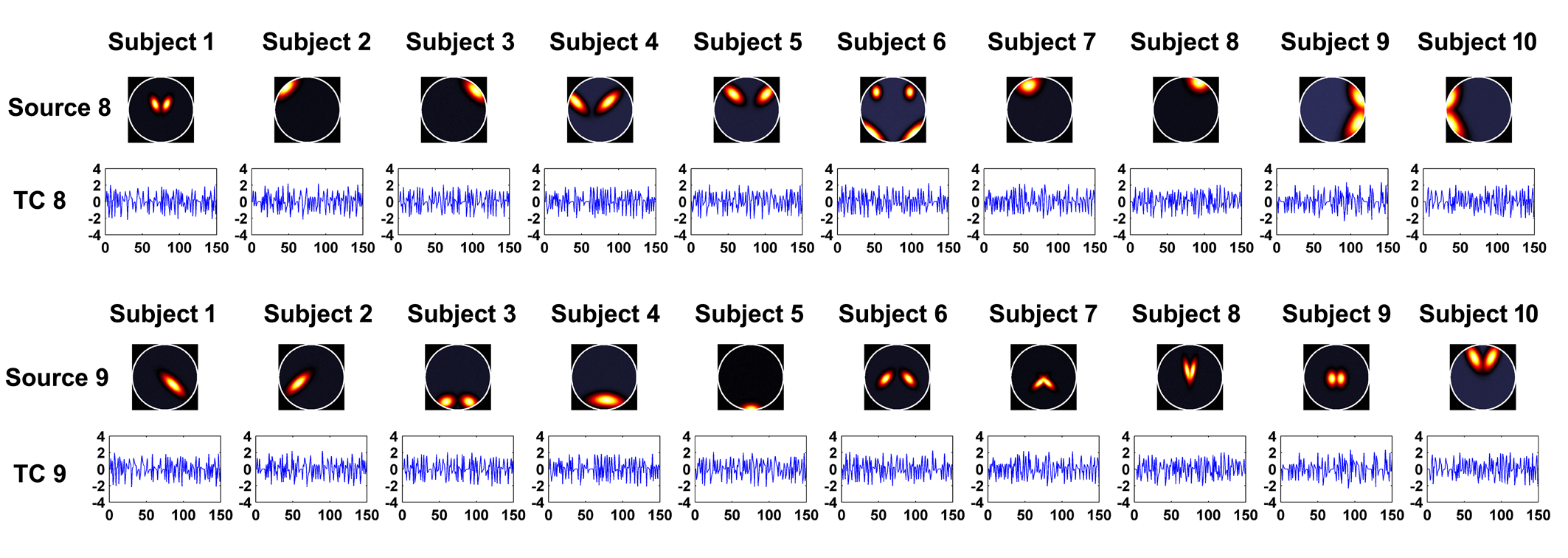
**

Fig. S3. The simulated two subject-unique sources (the 8^th^ source and the 9^th^ source) and related TCs of all subjects in supplementary Experiment 2.

**
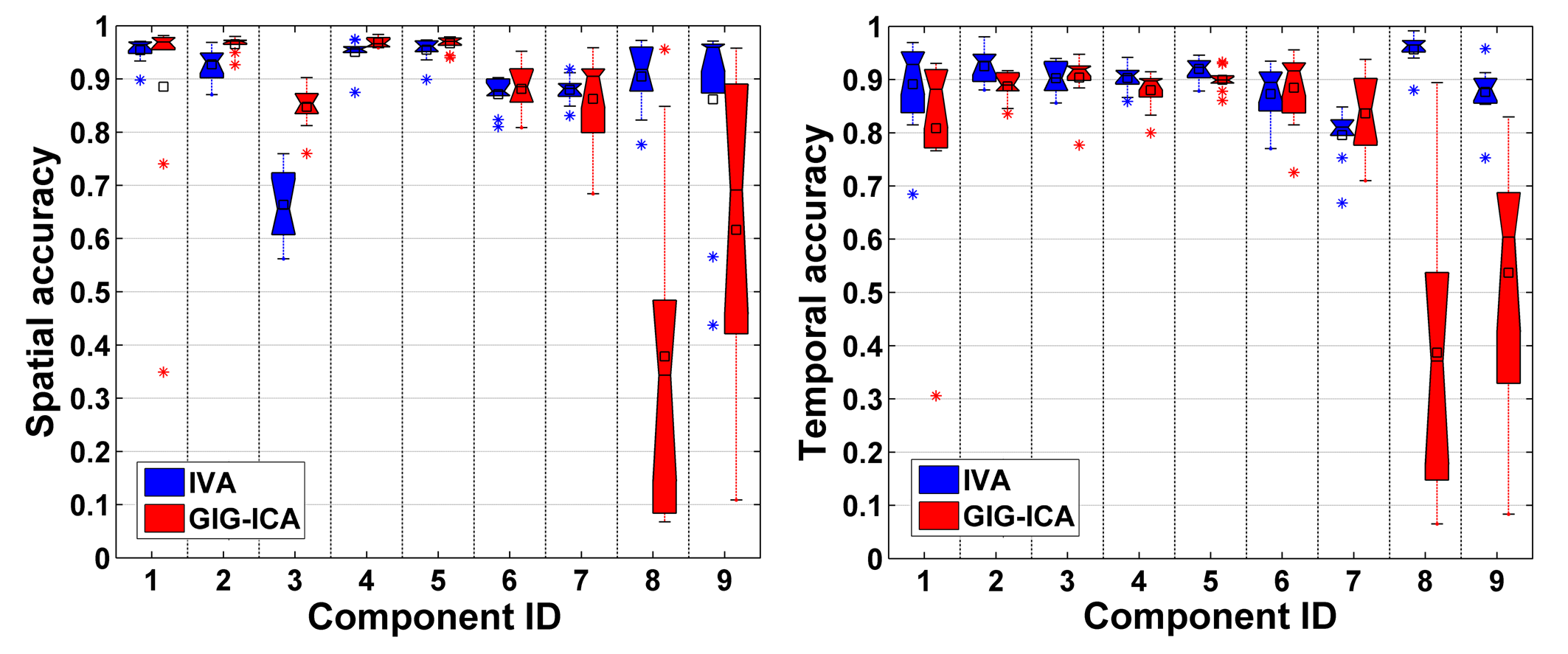
**

Fig. S4. Results of supplementary Experiment 2. Spatial and temporal accuracy of each estimated component and TC obtained from IVA and GIG-ICA for datasets with subject-unique sources. The 8^th^ and 9^th^ component are subject-unique. The y-axis denotes the spatial/temporal correlation between one subject-specific component/TC and the corresponding ground truth source/TC. The accuracy metrics of each component/TC from all subjects are shown using one boxplot. Each point in one boxplot corresponds to the spatial/temporal accuracy of one component/TC for one subject.
